# The therapeutic futility paradox: insights from oncological drug litigation in Ecuador

**DOI:** 10.3389/fmed.2025.1434524

**Published:** 2025-03-14

**Authors:** María Belén Mena Ayala, Xavier Maldonado

**Affiliations:** Universidad Central del Ecuador, Quito, Ecuador

**Keywords:** Ecuador (country), cancer, therapeutic futility, judicialization of health, right to health (source: MeSH NLM), pharmacoepidemiogy, drug utilisation studies

## Abstract

**Background:**

In oncology, patients with advanced cancer are often subjected to treatments with limited therapeutic value. This phenomenon is amplified through drug litigation, where interpretations of the right to life and health can lead to decisions that fail to adequately consider evidence of real benefits.

**Methods:**

This descriptive study analyzed discrepancies between key arguments in judicial rulings that favored access to oncological drugs and the outcomes of related clinical trials. We reviewed 5 rulings issued in Ecuador between 2012 and 2018 that represented claims from 36 patients. The analysis focused on comparing judicial decision arguments against evidence from pivotal clinical trials regarding quality of life and overall survival.

**Results:**

The 16 litigated drugs were approved through accelerated pathways, of which 37.5% were classified by the European Medicines Agency (EMA) as requiring additional monitoring. While 97% of rulings stated that the litigated drugs improved quality of life or survival, clinical trials reported favorable benefits in less than 20% of cases for the judicially contested indications.

**Conclusion:**

These findings reveal significant discrepancies between available scientific evidence and the arguments supporting judicial decisions in cases involving access to oncological drugs in Ecuador.

## Introduction

### Hope or false hope

‘Therapeutic futility’ refers to the application of treatments or interventions that do not produce significant benefits in terms of overall survival or disease remission, despite the associated risks and side effects (1).

Cancer drugs play a crucial role in cancer treatment. However, sometimes, the line between hope and false hope offered to patients becomes blurred, particularly when several lines of treatment have failed or the patient is no longer eligible due to the progression of the disease; then, the provision of the drug becomes a futile option (1, 2).

As a society we can assume that the drugs that circulate in the oncology pharmaceutical market prolong life and bring well-being, autonomy, comfort, joy to the patient; that is, quality of life. However, the reality is different since this is not an essential requirement for medicines to reach the market. In cancer treatment, survival is meaningless if it is not directly related to quality of life (3).

This raises questions about what the current priorities are in drug development and regulation (4–7).

In 2012, the United States Food and Drug Administration (U.S. FDA) made a significant decision to withdraw a breast cancer medication from the market after determining it lacked therapeutic value. Dr. Mikkael A. Sekeres, who served on the committee evaluating this case, offered a poignant perspective that highlighted the ethical implications of this decision (8). As a member of the U.S. FDA Oncologic Drugs Advisory Committee, Dr. Sekeres’ insights provided a crucial window into the complex decision-making process surrounding cancer drug approvals. His statement would go on to frame a critical discussion about the balance between providing hope and avoiding false promises in cancer treatment. He said he said something like:

*"We try to be dispassionate, but we always think about the person in front of us in the clinic, sitting a foot or two away from us in our cramped exam rooms, waiting to hear what treatment we can offer to get rid of their cancer. What kind of conversation would you have with such a patient if you were trying to convince her to accept a treatment like this? "Well, I can offer you a drug that won't make you live longer, won't make you feel better, and can have life-threatening side effects, but it will keep your cancer from getting worse for an average of 1 to 2 months.*

*Hope? Or false hope?”*

### Accelerated drug approval, between and futility

In 1992, the U.S. Congress authorized the U.S. FDA to create an accelerated approval pathway to help develop new drugs intended to treat serious or life-threatening diseases and thereby provide a significant advantage over available therapies. In this way, the U.S. FDA can approve drugs by demonstrating an effect on an intermediate clinical endpoint (surrogate variable or surrogate variable) that is “reasonably likely” or that could predict an actual clinical endpoint, such as changes in symptoms or mortality rates (9, 10).

These intermediate endpoints typically include response rate, progression-free survival, time to tumor progression, time from randomization to tumor progression (not including deaths), invasive disease-free survival, and pathologic complete response rate (11). Regulatory agencies generally do not require clinical trials that include clear evidence of improved quality of life or patient autonomy.

Many of the drugs that have received accelerated approval do not show a clear clinical benefit in subsequent confirmatory trials. In this regard, a study that evaluated 46 oncological indications for drugs with more than 5 years of follow-up, revealed that once on the market, less than half of these drugs (43%) demonstrated a clinical benefit in subsequent trials. This represents therapeutic futility despite regulatory marketing approval (12).

It should be recognized that the accelerated approval mechanism, while favoring access to medicines where there are therapeutic gaps, also implies the use of medicines with incomplete safety profiles.

Some medicines that do not show benefit in post-approval trials despite their futility often remain on the market or in clinical guidelines. One study found that 61% of cancer drugs with negative results in post-accelerated approval trials remained on clinical guideline indications and recommendations several years after they were shown to provide no improvement in the primary efficacy endpoint (13).

### Regulatory reliance and its implications

Regulatory reliance is a mechanism by which health agencies use and rely on regulatory decisions from other jurisdictions to streamline their own approval processes based on the robustness of their standards and scientific processes. In Latin America and the Caribbean, this practice has become widespread: 13 out of 20 regulatory authorities have adopted this mechanism, recognizing or shortening their approval processes when a drug has already been authorized by reference agencies such as the European Medicines Agency—EMA and U.S. FDA.

This mechanism, although it seeks to optimize resources and speed up access to medicines, has critical gaps. We homologate accelerated approvals, requiring subsequent confirmatory studies, additional follow-up with pharmacovigilance plans. Studies that are never done in countries that adopt regulatory reliance (9, 12).

The case of Ecuador illustrates these limitations: its approval system allows drugs approved by high health surveillance agencies to automatically obtain sanitary registration, without discriminating whether they were approved by accelerated pathways or require additional monitoring. This incomplete reliance results in the approval of products for sale without replicating the surveillance conditions necessary to guarantee their safety in real scenarios (14).

### Judicialization in access to medicines

Access to medicines is a fundamental part of the right to health. This implies significant efforts for Health Systems to guarantee availability and access in an equitable and sustainable manner. When this is not achieved; and, given that rights are judicially enforceable, in some Latin American countries, judicialization to access a medicine or health benefit is the way. A path that grows exponentially (15).

Judges end up resolving these dilemmas, attached to their legal interpretation of the right to health. On the one hand, they must protect the constitutional right from a potential threat to health, dignity and life; on the other hand, they lack the technical knowledge necessary to evaluate the scientific evidence on the efficacy or futility of the requested treatments. Judicialization can distort public health priorities (16, 17).

Cancer patients, facing potentially serious prognoses, seek therapeutic options, even those with marginal or unproven benefits. This phenomenon is amplified by the tendency to overestimate the potential benefits of new therapeutic schemes, creating what some authors call “therapeutic mirage” (18–20).

The literature indicates that a large part of the cancer drugs prosecuted are approved through accelerated pathways, using surrogate variables that do not necessarily translate into clinically significant benefits or quality of life. This phenomenon can lead to the prescription of treatments of questionable therapeutic value in the name of the right to health (21).

A particularly worrying aspect is the role of prescribing physicians in this process. There is evidence that some health professionals may overestimate the benefits of new treatments or be influenced by the pharmaceutical industry, contributing to the prosecution of drugs of marginal benefit. This phenomenon is amplified when their testimonies in court are taken as definitive evidence, without contrasting with data from clinical trials or systematic reviews (22).

The consequences of this process go beyond the economic impact. Judicialization can create a parallel system of access to medicines that undermines the principles of systematic health technology assessment and rational use of resources (23).

The situation becomes even more complicated when we consider that many of these drugs, in addition to having marginal benefits, can expose patients to significant adverse effects as documented by the Constitutional Court of Ecuador, revealing that there are cases where judicialization has led to the use of drugs that are not only futile but potentially dangerous (24).

### Access to medicines in Ecuador

Ecuador considers access to safe and effective quality medicines as part of the right to health, access to medicines with public resources is governed by the (25) National Table of Basic Medicines (*Cuadro Nacional de Medicamentos Básicos*), a list periodically updated by the National Health Council that is built following the guidelines of the World Health Organization and selects essential medicines based on efficacy criteria, safety, convenience and profitability (26) seeking to meet the health needs of the majority of the population.

For drugs not included in this list, Ecuador has established specialized committees that analyze individual cases. They evaluate each application based on scientific evidence of quality, safety and efficacy, their decisions are binding and oblige the State to guarantee access to approved medicines, seeking to balance individual needs with broader public health considerations actions (27–30). However, it has been documented that, when these committees deny access to medicines due to poor efficacy or because the patient is no longer eligible, some patients resort to judicialization, seeking access through constitutional legal (31–33).

This descriptive study analyzed the discrepancies between the decisive arguments of court rulings that favored access to cancer drugs and the results of related clinical trials. It reviewed 25 judgments issued in Ecuador between 2012 and 2018 that represented the claims of 36 patients. The analysis focused on three main variables: quality of life, overall survival, time free of disease progression, contrasting judicial decisions with data from pivotal clinical trials in the judicialized indications.

### Methodology

This descriptive study analyzed the precautionary measures filed against the Ministry of Public Health of Ecuador between 2012 and 2018 related to access to oncology medication. The search for judicial processes was carried out on the website of the Judicial Branch of Ecuador,^1^ specifically in the category “Protection Action.”

Cases where the Ministry of Public Health was sued for access to oncology drugs during the period 2012–2018 were selected. This period was chosen because it coincides with the issuance of regulations aimed at improving access to drugs that are not considered essential, through the implementation of committees of experts for the analysis of individualized cases (34, 35).

To evaluate the scientific evidence related to the prosecuted drugs, data on quality of life, time free of disease progression and overall survival were extracted from the technical data sheets established by the EMA and the U.S. FDA. The use of these sources responded to the absence of equivalent information in the National Agency for Health Regulation, Control and Surveillance of Ecuador (ARCSA) and to the homologation mechanisms covered by the regulatory reliance between agencies (36).

The study was approved by the Ethics Committee of the Central University of Ecuador (Code 0013-FCM-D-2019) and all data was anonymized to protect the privacy of the people involved in the legal proceedings.

## Results

This study’s analysis encompassed 26 judicial proceedings involving 114 patients who sought access to oncological medications through legal channels in Ecuador between 2012 and 2018. Among these proceedings, 21 were individual claims, while 5 were collective actions representing multiple patients.

**Figure 1:**
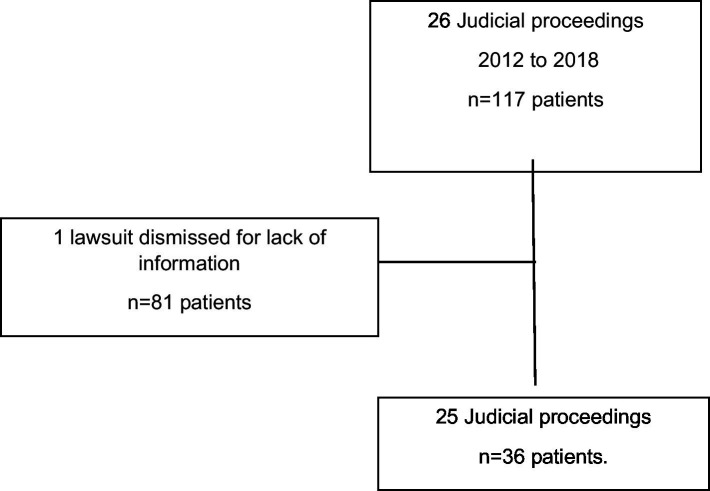
Court procedure selection flowchart.

One collective proceeding involving 81 patients was excluded due to inconsistent documentation, resulting in a final analytical sample of 25 proceedings representing 36 oncological patients (Figure 1).

The demographic analysis revealed that 60.6% of patients were male, with a mean age of 74 years, while female patients (39.4%) had a mean age of 56 years. The most frequent cancer types were hematological malignancies (48.5%), followed by prostate cancer (18.2%), and lung cancer (15.2%). Less frequent cases included skin and kidney cancer (6.1% each), and breast and colon cancer (Table 1).

**Table 1:** General characteristics of patients seeking judicial access to oncological drugs in Ecuador (2012–2018).

Characteristic	Percentage (%)	Mean Age (years)
Gender		
Male	60.6	74
Female	39.4	56
Affected organ		
Oncohematology	48.5	29
Prostate	18.2	78
Lung	15.2	48.5
Skin	6.1	58
Kidney	6.1	74.5
Breast	3.0	62
Colon	3.0	60

The analysis identified 16 distinct oncological medications that were the subject of litigation. These medications were primarily indicated for hematological disorders (48.5%) and metastatic, unresectable, or first-line treatment-resistant cancers (51.5%).

Notably, all medications (100%) had received accelerated approval from reference regulatory agencies (US FDA, EMA). Among these medications, 37.5% (6/16) carried the EMA’s black triangle symbol (▼) indicating additional monitoring requirements under the European pharmacovigilance system. While U.S. FDA’s accelerated approval program inherently requires post-marketing confirmatory trials for all approved drugs, it does not use a specific additional monitoring designation. Only one medication lacked regulatory approval in Ecuador at the time of litigation (Table 2).

**Table 2:** Characteristics of judicially requested medications.

Characteristic	Number (*n* = 16)	Percentage (%)
Reference agency approval path (U.S. FDA/EMA)		
Accelerated approval	16	100
Regular approval	0	0
EMA additional monitoring		
Required	6	37.5
Not required	10	62.5
Registration status in Ecuador		
Registered	15	93.8
Not registered	1	6.2

**Figure 2:**
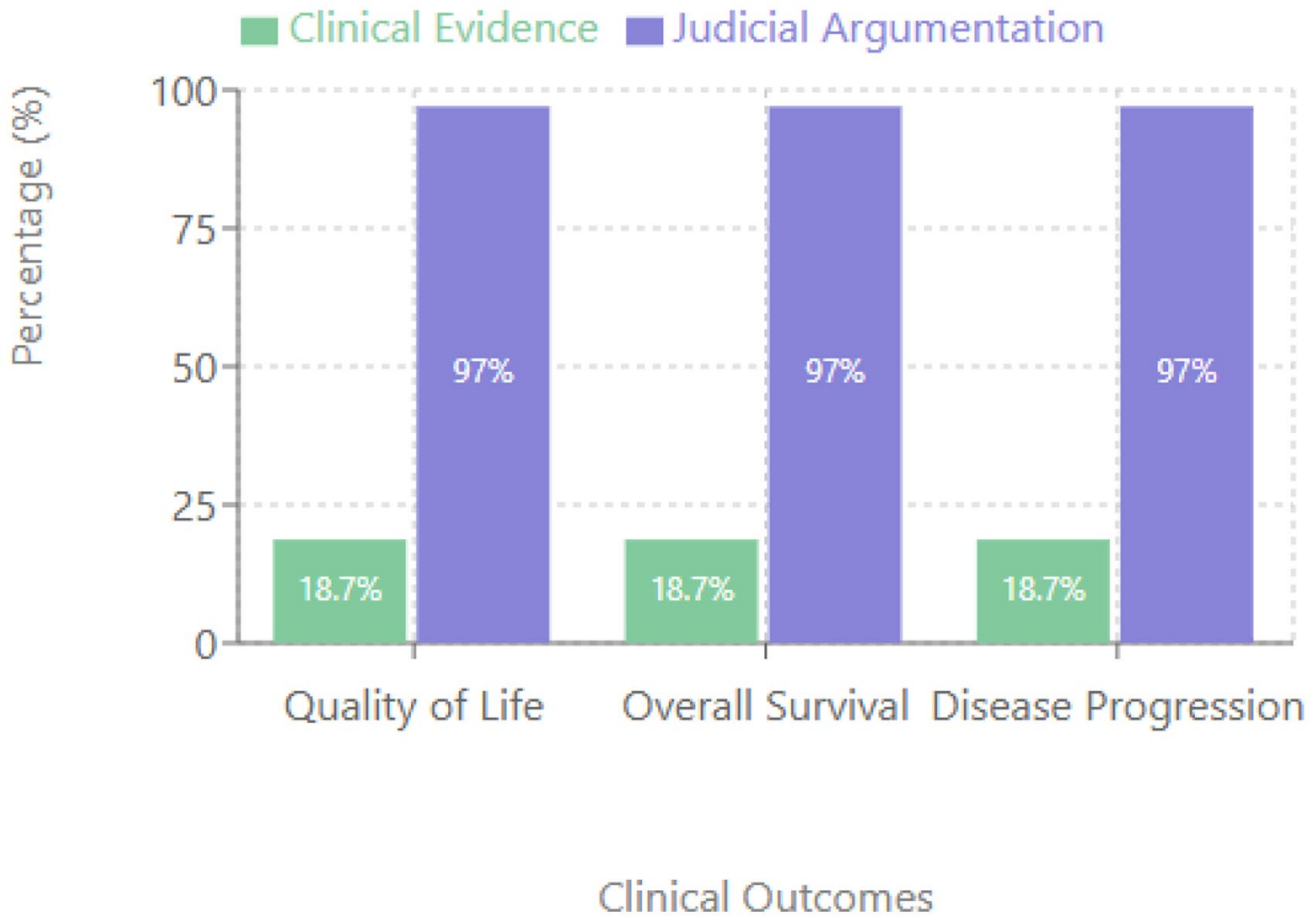
Comparison between clinical evidence and judicial argumentation.

A striking disparity emerged between clinical evidence and judicial decisions (Figure 2).

While 97% of judicial rulings cited quality of life improvement and life extension as decisive arguments, only 18.7% of the medications demonstrated such benefits in pivotal clinical trials for the litigated indications.

This discrepancy was consistent across all evaluated outcomes: quality of life improvement, overall survival extension, and progression-free survival of ≥6 months.

In 97% of cases, judges based their decisions primarily on the prescribing physician’s testimony, particularly regarding two critical assertions: (1) the risk of death if the medication was denied, and (2) the potential improvement in quality of life if access was granted. This reliance on physician testimony persisted even when it contradicted available clinical evidence.

These findings highlight a significant gap between scientific evidence and judicial decision-making in cases involving access to oncological medications in Ecuador during the study period. The data suggest that judicial decisions were more heavily influenced by physician testimony than by documented clinical evidence from pivotal trials.

## Discussion

The findings of this study indicate significant discrepancies between the benefits claimed in court and the available scientific evidence. As evidenced in the results, 97% of the sentences claimed improvements in quality of life or survival, while only 18.7% of the drugs showed such benefits in the pivotal trials for the judicialized indications. This gap reflects a deeper paradox that begins with the accelerated approval of drugs with marginal benefits (13).

### Incomplete regulatory reliance

Our findings indicate that the litigation was not primarily due to lack of approval in Ecuador, as 93.8% of the drugs already had sanitary registration at the time of the litigation. The problem lies in the fact that the approval system does not distinguish whether the drugs were approved through accelerated pathways or require additional follow-up. This incomplete “reliance” allows marketing without replicating the surveillance conditions necessary to guarantee safety in real scenarios, especially when the patient is practically being administered the drug by a judge (3, 36).

### Discrepancies in reported benefits

The stark difference between benefits claimed in court and scientific evidence is troubling. While 97% of the judgments cited improvements in quality of life or survival as decisive arguments, only 18.7% of the drugs demonstrated such benefits in pivotal trials. This discrepancy suggests a potential systematic misinformation that influences judicial decisions.

### Ethical and practical implications

The exaggeration of benefits in judicial contexts raises serious ethical concerns. As evidenced by the Constitutional Court of Ecuador in Judgment No. 679-18-JP/20 (33), there were cases where the drugs could cause unacceptable toxicity and the judges allowed the administration of these drugs that were not only futile but dangerous. This reality suggests that the justice system is making decisions based on expectations not supported by scientific evidence, possibly compromising the well-being of patients.

It is critical that healthcare professionals and decision-makers provide accurate information about the efficacy and potential risks associated with the use of these medicines, avoiding exaggerations that may lead to false expectations (22).

### The influence of medical judgment on judicial decisions

Our analysis reveals that in 97% of the sentences, the judge based his decision on the criterion of the prescribing physician as an expert word, specifically with respect to two arguments: the possibility of death if the drug is denied and the improvement in the quality of life if access is approved. This dynamic, far from being an isolated phenomenon, reflects a pattern documented in the literature. For example, a study in Brazil found close collaboration between prescribing physicians and lawyers in drug lawsuits, where a single doctor came to be responsible for 16.5% of prescriptions for a specific drug, all funneled through a single law firm. While it is essential that prescribing physicians are heard in judicial proceedings for their expertise, it is worrying that their testimonies are not contrasted with the available scientific evidence or that potential conflicts of interest are examined. The normalization of this practice, where a health professional can defend before a judge the use of a drug with unproven benefits and receive absolute credibility, undermines the principles of elementary decency of the medical profession and compromises the well-being of patients (37).

The judicialization of access to medicines, although it appears to protect individual rights, promotes a therapeutic mirage where judicial decisions, by prioritizing individual cases over scientific evidence, can undermine both individual and collective well-being. This phenomenon not only represents a tension between individual and collective rights, but also reveals a deeper problem: the granting of access to medicines of unproven benefit generates false hopes and compromises resources that could be allocated to interventions of proven benefit, thus threatening the sustainability and equity of public health policies.

We firmly believe that everyone has the fundamental right to timely access to quality, safe and effective medicines. We also maintain that every person has the right to a dignified life, which should not be subject to the provision of a futile medicine. It is important to recognize that some medications fail to improve quality of life, a reality that seems to be unknown to some judges, lawyers, health professionals or even patients. When questioning useless prescriptions, it is not simply a matter of economic considerations or patient pressure, but of avoiding false hope and ensuring the integrity and safety of health care.

### Corollary

The judicial route represents a legitimate tool to guarantee the right to health when other mechanisms have failed. However, our study reveals troubling discrepancies that require urgent attention (24).

It is ethically questionable that terminally ill patients should spend their final days in legal battles, pursuing unfounded hopes.

Judges are not antagonists in this narrative. They are, possibly, additional victims of a system that allows the marketing of drugs of little therapeutic value and tolerates the exaggeration of benefits by some health professionals. We are all victims of this systemic dysfunction until health is addressed in a comprehensive way, until the uncertainty inherent in treatments is honestly communicated, until their situation is explained to patients in understandable terms, and until health systems prioritize both a dignified life and a dignified death (38).

## Conclusion

This study reveals significant discrepancies between scientific evidence and judicial decisions related to access to oncology drugs in Ecuador. The findings are troubling: 97% of the judgments cited improvements in quality of life and survival as decisive arguments, while only 18.7% of the drugs demonstrated such benefits in pivotal trials. This gap suggests systematic misinformation in the judicial process.

The analysis shows that the problem does not lie in the lack of regulatory approval—93.8% of the drugs already had a sanitary registration at the time of their prosecution. Rather, it exposes the consequences of an incomplete regulatory reliance that allows the commercialization of approved drugs on an accelerated basis without replicating the necessary surveillance conditions.

The determining influence of medical judgment on judicial decisions (97% of cases) without contrast with the available scientific evidence, together with the lack of evaluation of conflicts of interest, suggests the urgent need to reformulate how scientific evidence is incorporated in judicial processes related to access to cancer drugs.

We have a critical need to strengthen the integration between regulatory systems, clinical practice, and judicial processes to ensure that decisions on access to medicines are based on sound scientific evidence, thus protecting both the right to health and the safety of patients.

## Data Availability

The datasets presented in this study can be found in online repositories. The names of the repository/repositories and accession number(s) can be found at: https://procesosjudiciales.funcionjudicial.gob.ec/busqueda.

## Footnotes

^1^ https://procesosjudiciales.funcionjudicial.gob.ec/busqueda
